# Association of *Fatty Acid Desaturase 1* rs174547 Polymorphism with the Composition of Long-Chain Polyunsaturated Fatty Acids in Serum Glycerophospholipids during Pregnancy

**DOI:** 10.3390/nu15030722

**Published:** 2023-01-31

**Authors:** Terue Kawabata, Hideoki Fukuoka, Michiru Harada, Kumiko Shoji, Yoshinori Kubo, Chisato Mori, Kenichi Sakurai, Takeshi Ohkubo, Kyoichi Oshida, Yuichiro Yamashiro

**Affiliations:** 1 Faculty of Nutrition, Kagawa Nutrition University, 3-9-21 Chiyoda, Sakado 350-0288, Japan; 2 Department of Perinatal Mesenchymal Stem Cell Research, School of Medicine, Fukushima Medical University, 1 Hikarigaoka, Fukushima 960-1295, Japan; 3 Department of Bioenvironmental Medicine, Graduate School of Medicine, Chiba University, 1-8-1 Inohana, Chuo-ku, Chiba 260-8670, Japan; 4 Department of Sustainable Health Science, Center for Preventive Medical Sciences, Chiba University, 1-33 Yayoi-cho, Inage-ku, Chiba 263-8522, Japan; 5 Department of Nutrition and Metabolic Medicine, Center for Preventive Medical Sciences, Chiba University, 1-33 Yayoi-cho, Inage-ku, Chiba 263-8522, Japan; 6 Department of Health Nutrition, Faculty of Human Sciences, Sendai Shirayuri Women’s College, 6-1 Honda-Cho, Izumi-ku, Sendai 981-3107, Japan; 7 Department of Pediatrics, Juntendo University, 3-1-3 Hongo, Bunkyo-ku, Tokyo 113-8431, Japan; 8 Probiotics Research Laboratory, Graduate School of Medicine, Juntendo University, 2-9-8-3F, Hongo, Bunkyo-ku, Tokyo 113-0033, Japan

**Keywords:** docosahexaenoic acids, trimester, pregnancy, *fatty acid desaturase 1*

## Abstract

The increase in fetal requirements of long-chain polyunsaturated fatty acids (LCPUFAs) during pregnancy alters maternal fatty acid metabolism, and therefore, fatty acid desaturase (*FADS*) gene polymorphisms may change blood fatty acid composition or concentration differently during pregnancy. We investigated the relationship between a *FADS1* single-nucleotide polymorphism (SNP) and maternal serum LCPUFA levels in Japanese pregnant women during the first and third trimesters and at delivery. Two hundred and fifty-three pregnant women were included, and fatty acid compositions of glycerophospholipids in serum (weight %) and the *FADS1* SNP rs174547 (T/C) were analyzed. LCPUFAs, including arachidonic acid (ARA) and docosahexaenoic acid (DHA), significantly decreased from the first to the third trimester of pregnancy. Furthermore, DHA significantly decreased from the third trimester of pregnancy to delivery. At all gestational stages, linoleic acid (LA) and α-linolenic acid were significantly higher with the number of minor *FADS1* SNP alleles, whereas γ-linolenic acid and ARA and the ARA/LA ratio were significantly lower. DHA was significantly lower with the number of minor *FADS1* SNP alleles only in the third trimester and at delivery, suggesting that genotype effects become more obvious as pregnancy progresses.

## 1. Introduction

**Figure 1:**
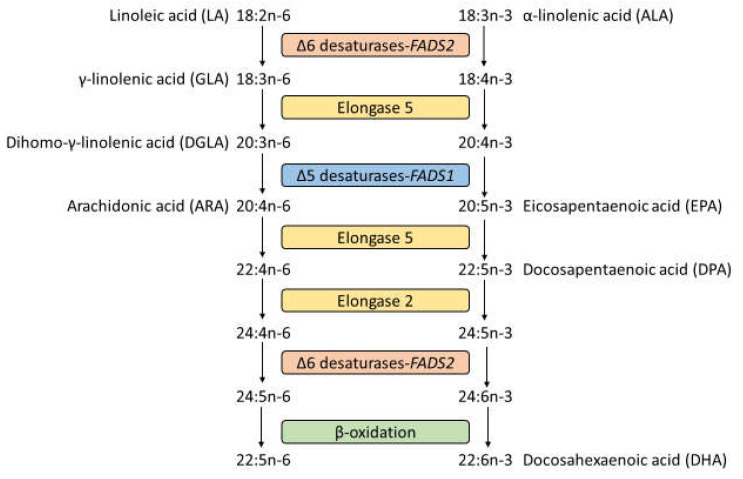
The n−6 and n−3 polyunsaturated fatty acid metabolism pathways.

The long-chain polyunsaturated fatty acids (LCPUFAs), arachidonic acid (ARA) (20:4n−6), and docosahexaenoic acid (DHA) (22:6n−3), are endogenously synthesized by Δ-5 and Δ-6 desaturases and elongase 2 and 5 enzymes from precursor PUFAs [1] (Figure 1). Single-nucleotide polymorphisms (SNPs) were identified in fatty acid desaturase genes 1 and 2 (*FADS1* and *FADS2*, respectively), encoding Δ-5 and Δ-6 desaturase, respectively, and the elongation of very long chain fatty acids genes 2 and 5 (*ELOVL2* and *ELOVL5*, respectively), encoding elongase 2 and 5, respectively [2]. The effects of *FADS1*, *FADS2*, and *ELOVL2* SNPs on blood fatty acid composition and concentration have been reported [3,4,5].

*FADS1* resides on chromosome 11q12–q13.1, and the SNP rs174547 (T/C) located in intron 9 of *FADS1* regulates FADS1 (or Δ-5 desaturase) expression [2,6]. Nakayama et al. reported that the copy number of the minor allele for the SNP rs174547 was significantly associated with increased blood triglyceride levels and decreased high-density lipoprotein cholesterol levels in approximately 20,000 Japanese subjects [6]. We administered ^13^C-linoleic acid (LA) (18:2n−6) to healthy young and elderly subjects and found that the area under the curve of the concentration of ^13^C-ARA, the metabolite of LA, was 57% in heterozygotes (TC) and 37% in minor homozygotes (CC) compared with major homozygotes (TT) for rs174547 [7], directly indicating that the endogenous synthetic capacity of LCPUFAs is suppressed in the variant of the gene polymorphism. Furthermore, in an observational study involving an elderly Japanese population, we found that rs174547 C-minor allele carriers had significantly higher LA and lower ARA in erythrocyte membrane and plasma phospholipids (15% and 6% ARA reduction, respectively, per C-allele) [8]. A Dutch study using plasma cholesterol ester and a Korean study using plasma have shown results similar to ours [9,10]. An Australian study showed elevated erythrocyte LA in rs174547 C-allele carriers [11]. For the Japanese, Western, and Asian populations, *FADS1* rs174547 may be an important SNP affecting fatty acid metabolism.

The role of n−3LCPUFAs during gestation is to reduce the risk of preterm birth [12,13] and to contribute to the neural and visual development of the fetus [14]. The n−3LCPUFAs required for these roles are supplied either by direct uptake from the maternal diet or by endogenous synthesis from precursor PUFAs [1]. Therefore, the effect of *FADS* gene polymorphisms that affect the endogenous synthesis of LCPUFAs in women during pregnancy should be elucidated. We previously analyzed the *FADS1* SNP rs174547 in 383 pregnant Japanese women with gestational ages of 24–30 weeks and found higher LA and significantly lower ARA and DHA, converted products, in minor C-allele carriers than those in major T-allele homozygotes [15]. Thus, the rs174547 polymorphism is an important factor in determining blood PUFA composition in Japanese pregnant women.

The fetal LCPUFA requirements increase as pregnancy progresses, and maternal fatty acid metabolism changes markedly [16]. Therefore, the effect of *FADS* gene polymorphisms on blood fatty acid composition or concentration may change throughout the gestational period. Longitudinal gestational changes in the relationships between FADS gene polymorphisms and blood fatty acid compositions in mothers were investigated in a US study (at 14.5 weeks’ gestation and birth) [17] and a Canadian study (at 16 and 36 weeks’ gestation) [18]. In the US study, minor allele homozygotes for *FADS1* rs174533 had significantly lower erythrocyte DHA and ARA status at 14.5 weeks’ gestation (mean) and significantly lower maternal erythrocyte DHA at birth than major-allele carriers; however, no significant difference in maternal erythrocyte ARA was observed at delivery [17]. In the Canadian study, no difference in the 20 and 22 carbon chain n−3 fatty acids/ALA ratio in erythrocyte ethanolamine phosphoglyceride at 16 weeks’ gestation was observed between the major-allele carriers and minor allele homozygotes of *FADS1* rs174553; however, this ratio was significantly lower in minor allele homozygotes at 36 weeks’ gestation [18]. Thus, the effect of the *FADS* polymorphism on maternal blood fatty acid status probably differs between the first trimester and late pregnancy or at birth; however, data available to us are very limited and have not led to a unified view.

Therefore, we longitudinally examined how the *FADS1* gene polymorphism rs174547 affects LCPUFAs in maternal serum glycerophospholipids during pregnancy (12 weeks in the first, 32 weeks in the third trimester of pregnancy, and at delivery) in Japanese pregnant women living in areas of adjoining Tokyo.

## 2. Materials and Methods

### 2.1. Ethics Approval

The study protocol was approved by the Biomedical Research Ethics Committee of the Graduate School of Medicine, Chiba University (ID: 989, 20 September 2019), the Ethics Review Committee for Human Genome/Gene Analysis Research, Waseda University (ID: 2013-G002 (3), 13 November 2015), the Medical Ethics Committee of Kagawa Nutrition University (ID: 67, 20 July 2016; ID:284-G, 15 July 2020), and was conducted according to the Declaration of Helsinki. All participants gave informed consent for inclusion before participating in the study.

### 2.2. Study Population

This study was conducted as a part of the Chiba Study of Mother and Child Health (C-MACH) at the Center for Preventive Medical Sciences, Chiba University, and the Research Institute for Science and Engineering, Waseda University [19]. The C-MACH consists of three hospital-based cohorts from the Onodera Ladies Clinic and the Yamaguchi Women’s Hospital, in the Chiba Prefecture and the Aiwa Hospital in Saitama Prefecture. The Chiba and Saitama prefectures are adjacent to Tokyo, the largest city in Japan. The participants were recruited between February 2014 and June 2015. The recruitment population consisted of pregnant women examined at <13 weeks of gestation in the three hospitals. The study population included children born to women who consented to participate. If a stillbirth occurred, the woman’s participation in this study was terminated. Consent to participate in C-MACH was obtained from 433 women. Twenty-five women withdrew from the study after providing informed consent, resulting in a final cohort of 408 women [19]. The Onodera Ladies Clinic and Aiwa Hospital cohorts of the C-MACH were used for this study. Only the participants whose sera were collected at the time of recruitment and those who completed the maternal *FADS1* SNP analysis were included in the study, resulting in a final cohort of 242 pregnant women.

### 2.3. Maternal and Infant Information

Self-administered lifestyle questionnaires for the mothers were administered at the first (12 weeks) and third (32 weeks) trimesters of pregnancy. Maternal age and parity and non-pregnant height and weight were obtained from the early pregnancy questionnaire. Body mass index (BMI) was calculated from height and weight. The smoking status was obtained from the questionnaires administered during the first and third trimesters of pregnancy. Information on the sex of the child and gestational age was obtained from the medical records at delivery.

### 2.4. Dietary Survey

The mothers completed a brief-type self-administered diet history questionnaire (BDHQ) at 12- and 32-weeks’ gestation. The validity of the BDHQ was confirmed previously using the 16-day weighted dietary record as the gold standard [20]. In the data analysis, various fatty acid intakes were used in the analysis as energy ratios. The exclusion criteria for reporting errors on the BDHQ were values < 0.5 times the energy requirement of Physical Activity Level I or >1.5 times the energy requirement of Physical Activity Level III for each subject.

### 2.5. Fatty Acid Analysis in Serum Glycerophospholipids

Blood samples were collected in the first and third trimesters of pregnancy (12- and 32-weeks’ gestation, respectively) and at delivery. After collection, blood was separated into serum and clots, and the serum was frozen at −80 °C. The serum stored at the Center for Preventive Medical Sciences, Chiba University, was then transferred to Kagawa Nutrition University using a cold storage box containing dry ice and continued to be frozen at −80 °C until fatty acid analysis. Serum fatty acid analysis was performed following the method of Glaser et al. [21]. Proteins were removed from the serum using methanol, followed by methyl ester exchange with sodium methoxide solution to produce fatty acid methyl esters in glycerophospholipids, and then, fatty acid analysis was performed by gas chromatography. The details are described in a previous report [22]. The ratio of the peak area of each fatty acid to the total peak area (weight %) was calculated and used as the value of fatty acids in serum glycerophospholipids.

### 2.6. Genotyping

For DNA extraction, we used concentrated blood. Concentrated blood, which is the remaining plasma dispensed, was stored at −80 °C at Chiba University and transferred to Kagawa Nutrition University. A fully automated nucleic acid extraction system (MagLEAD 12gC; Precision System Science, Matsudo-shi, Chiba, Japan) was used with the MagDEA Dx SV reagent (Precision System Science Co., Ltd., Matsudo-shi, Chiba, Japan). The rs174547 (an intronic T/C polymorphism of *FADS1*) genotypes of all participants were determined using a TaqMan probe with the 7500 Real-Time PCR System (Applied Biosystems, Foster City, CA, USA) at Kagawa Nutrition University.

### 2.7. Statistical Analysis

The data were checked for normality using normal distribution point plots. Variables determined to have non-normal distribution were transformed into the natural logarithm and subsequently used in the analysis. The agreement of genotype frequencies with Hardy–Weinberg equilibrium expectations was tested by the Chi-square test. Comparisons of fatty acid composition in serum glycerophospholipids according to stages of pregnancy were performed using repeated one-way analysis of variance, followed by the Tukey–Kramer honestly significant difference test for multiple comparisons between groups. To explore the association between fatty acid composition in maternal serum glycerophospholipids and genetic polymorphisms, multiple regression analysis was performed using the fatty acid composition as the objective variable and rs174547 as the explanatory variable, according to the stages of pregnancy. Genotypes were pre-transformed as an additive model according to the number of minor alleles (0 for major homozygotes and 1 for carriers of at least one minor allele). The corresponding fatty acid intake, mother’s age, mother’s non-pregnant BMI, and smoking were included in the analysis as covariates. Data on fatty acid intake and smoking status in the first trimester of pregnancy were used to analyze the fatty acid composition in the first trimester of pregnancy, and data on fatty acid intake and smoking status in the third trimester of pregnancy were used to analyze the fatty acid composition in the third trimester of pregnancy and at delivery.

All statistical analyses were performed using the JMP (SAS Institute Inc., Cary, NC, USA). Differences with *p*-values of less than 0.05 were considered statistically significant.

## 3. Results

### 3.1. Participant Characteristics

The characteristics of the subjects are shown in Table 1. For mothers, information on age at 12 weeks’ gestation, non-pregnant body size, parity, smoking status, and the *FADS1* genotype were listed, and for children, the gestational period, sex, and *FADS1* genotype were listed. Both genotypes for mother and child were in Hardy–Weinberg equilibrium (all *p* > 0.45).

### 3.2. Maternal Serum Fatty Acid Changes during Gestation

Fatty acid compositions of maternal plasma glycerophospholipids (weight % of each fatty acid to the total fatty acids) according to gestational age are shown in Table 2. From the first trimester to the third trimester of pregnancy, saturated fatty acids (SFAs), monounsaturated fatty acids (MUFAs), dihomo-γ-linolenic acid (DGLA) (20:3n−6), and α-linolenic acid (ALA) (18:3n−3) were significantly elevated, and PUFA, γ-linolenic acid (GLA) (18:3n−6), ARA, eicosapentaenoic acid (EPA) (20:5n−3), docosapentaenoic acid (DPA) (22:5n−3), DHA, ARA/LA ratio, and ARA/DGLA ratio were significantly decreased. Furthermore, from the third trimester of the pregnancy to delivery, DPA and DHA significantly decreased.

### 3.3. Associations between Serum Glycerophospholipid Fatty Acids and rs174547 Polymorphism

Table 3 shows the results of the multiple regression analysis with maternal serum glycerophospholipid fatty acid composition (%) as the objective variable and maternal rs174547 polymorphism as the explanatory variable. In all stages of pregnancy, LA and ALA were significantly positively associated with the number of minor rs174547 polymorphism alleles, whereas GLA and ARA and the ARA/LA ratio were significantly negatively associated. The standard beta values (Std β) for LA, ARA, and the ARA/LA ratio were particularly high (all >0.3 in absolute value; *p* < 0.001), indicating a strong association with the rs174547 genotype. DHA and the ARA/DGLA ratio showed no association in the first trimester of pregnancy; however, a significant negative association with the number of minor rs174547 polymorphism alleles was observed in the third trimester of pregnancy and at delivery. DGLA and EPA showed significant negative correlations or negative correlation trends with the number of alleles of the minor rs174547 polymorphism at all gestational stages.

## 4. Discussion

This study evaluated the association between fatty acid Δ5 desaturase gene polymorphism (*FADS1* rs174547) and fatty acid composition in maternal serum glycerophospholipids (weight % of each fatty acid to the total fatty acids) in the first and third trimester, and at delivery. As a result, minor allele carriers of the rs174547 polymorphism had significantly higher maternal serum n−6 LA and lower ARA than major homozygotes, throughout pregnancy. That is, minor allele carriers have elevated precursor fatty acids and lower product fatty acids, which are consistent with the previous observational studies, indicating that fatty acid desaturation is suppressed due to the decreased expression or activity of Δ5 desaturase of minor allele [8,15,23].

Several studies have examined the association between maternal *FADS* polymorphisms and blood PUFA composition or concentration during pregnancy [17,18,24,25,26,27,28,29,30,31]. Among them, one study examined the same *FADS1* rs174547 polymorphism as our study and reported the plasma phospholipid fatty acid composition in 180 pregnant women at 24 weeks’ gestation who participated in a Spanish birth cohort [24]. The results showed that the rs174547 C-minor allele count tended to be negatively correlated with the ARA/DGLA ratio, an index of Δ5 desaturase activity; however, the relationship was not significant, and the authors did not find a significant effect of the aforementioned SNP on pregnant women. In our study, we found a significant negative association between rs174547 C-minor allele counts and the ARA/DGLA ratio in late pregnancy (32 weeks) and at delivery; however, no association was observed in early pregnancy (12 weeks). Although the Spanish study and our study are not definitive because of the different timings of the analysis of the fatty acid composition in blood, our results agree with those of the Spanish study, demonstrating no association between the rs174547 polymorphism and the ARA/DGLA ratio at the relatively early stage of pregnancy.

In our study, the ARA/LA ratio showed a stronger correlation with the rs174547 genotype than the ARA/DGLA ratio. The Δ5 desaturase encoded by the *FADS1* gene converts DGLA to ARA. However, in this study, serum LA was higher, and fatty acids downstream from GLA were consistently lower in minor allele carriers than in major-allele homozygotes. This suggests that the metabolism of LA to GLA, which occurs upstream of the Δ5 desaturase point of action, is already suppressed. Similar results have been observed in our previous studies [7,8]. The rs174547 polymorphism is in linkage disequilibrium with many other *FADS* polymorphisms, including *FADS2* polymorphism [6,32]. Wang et al. also conducted a meta-analysis of the association between the rs174547 polymorphism and blood PUFAs and found that minor C-allele carriers were associated not only with reduced D5D activity index (ARA/DGLA) but also with reduced Δ6 desaturase activity index (GLA/LA) [33]. Therefore, regarding the effects of the *FADS1*/rs174547 SNP on plasma fatty acid composition, we believe that the ARA/LA ratio, which is an indicator of the activity of the entire fatty acid metabolic pathway, would more clearly confirm the association of plasma fatty acid composition with the *FADS1*/rs174547 SNP.

Several studies have reported a decrease in maternal blood DHA composition from approximately 18 weeks’ gestation to delivery [34,35,36,37]. We also found that the DHA composition in maternal erythrocytes during pregnancy was significantly lower at delivery than at 27 weeks’ gestation [38]. In this study, serum n−3LCPUFA composition, including DHA, also decreased from the first to delivery. In general, this change in maternal blood DHA composition in late pregnancy has been attributed to increased placental transfer of DHA from mother to fetus [16,34,39]. As a result, the demand for DHA in the mother increased from late pregnancy to delivery. The metabolism of ALA to EPA and DHA is regulated by negative feedback with the products of n−3LCPUFA [40], endogenous synthesis of DHA may be enhanced in late pregnancy and beyond, when demand for n−3LCPUFA increases. Notably, in this study, the association between *FADS1* polymorphisms and maternal serum DHA composition was found only in the third trimester and at delivery. We hypothesize that increased maternal demand for DHA may have increased endogenous synthesis of EPA and DHA from ALA, which would have resulted in more pronounced effects of genetic polymorphisms later in the pregnancy and beyond.

Another factor may contribute to the change in the relationship between *FADS1* polymorphisms and maternal serum DHA composition as pregnancy progresses. The total plasma lipid DHA composition is higher in women than in men [41], and the endogenous conversion of ALA to DHA has been reported to be greater in women than in men of the same age [42,43]. Furthermore, women taking oral contraceptives had approximately 10% higher DHA concentrations than those not taking them, and oral ethinyl estradiol, but not transdermal 17β-estradiol, increased DHA by 42% [44]. Oestrogen presumably upregulates DHA synthesis from ALA. Thus, it is more likely that the effects of SNPs affecting desaturase activity will be detectable after the second half of the pregnancy when oestrogen secretion increases.

In this study, we analyzed the association between fatty acid composition and genetic polymorphisms from as early as 12 weeks until delivery and examined the association between the fatty acid composition in the mother’s serum glycerophospholipids and genetic polymorphisms, including dietary fatty acid intake. However, this study has several limitations. In accordance with the previous study, we entered as covariates in multiple regression analysis only the fatty acid intake corresponding to each of the fatty acids as the objective variable, with the aim of excluding the effect of diet [45]. Because fatty acids are interrelated, it cannot be ruled out that fatty acids other than the corresponding fatty acid intake and blood fatty acids are related and that this may be a confounding factor. Furthermore, this is a hospital-based cohort study conducted in an urban setting within Japan, which limits the generalization of the results to areas with other different lifestyles.

Another limitation of this study was the evaluation of fatty acids in serum glycerophospholipids using relative composition (weight % of each fatty acid to the total fatty acids), not absolute concentration (mmol/mL or mg/mL). The plasma pool volume of pregnant women physiologically increases as pregnancy progresses. A meta-analysis showed that plasma volume increased beginning in the first week of pregnancy, with the steepest increase in the second trimester. Furthermore, plasma volume continued to increase during the third trimester. The pooled maximum increase in plasma volume was 1.13 L, resulting in a 45.6% increase in pregnant women compared with nonpregnant women [46]. Such changes in the plasma pool may significantly impact the interpretation of the results. For example, if the absolute amount of most fatty acids increases with increasing plasma pool volume, but the absolute amount of some fatty acids remains unchanged, the relative composition of these fatty acids will decrease. In other words, if there is an increase in the plasma pool volume during pregnancy, but the absolute amount of LCPUFA does not change while the absolute amount of other fatty acids increases, the relative composition of LCPUFA will decrease. Therefore, the absolute amounts may be misinterpreted as decreasing. The results of our study show a decrease in most LCPUFA compositions and fatty acid ratios (ARA/LA and ARA/DGLA) during pregnancy. However, this does not imply that the absolute amount of LCPUFA decreased. At this stage, we cannot discuss changes in absolute amounts of LCPUFA because we have not examined absolute concentrations or plasma-pooled amounts of fatty acids during pregnancy. Stark et al. demonstrated that fatty acid data vary depending on the relative composition, absolute concentration, and total amount in the plasma pool [45]. Therefore, fatty acid status during pregnancy is best reported in both relative composition and absolute concentration for proper interpretation. The results of this study are based on relative compositions. Thus, the use of this data is limited.

## 5. Conclusions

LCPUFAs, including ARA, DPA, and DHA, significantly decreased from the first to the third trimester of pregnancy. Furthermore, DPA and DHA significantly decreased from the third trimester of pregnancy to delivery. The association between n−6 PUFAs and the rs174547 genotype was strong at all gestational stages. DHA, an n−3 fatty acid, was associated with the rs174547 genotype only in the third trimester and at delivery, suggesting that genotype effects become more obvious as pregnancy progresses.

## Figures and Tables

**Table 1:** Subject characteristics.

Variables	n	(%)	Median	(25th–75th)
Mothers				
Age at participation				
<30 years old	63	(27.9%)		
30–39 years old	148	(65.5%)		
≧40 years old	15	(6.6%)		
Missing	16	-		
Height, cm	226		159	(155–163)
Prepregnancy weight, kg	226		52	(48–58)
Prepregnancy body mass index (kg/cm^2^)	226		20.7	(19.1–22.9)
Parity				
0	91	(40.8%)		
1	96	(43.1%)		
≧2	36	(16.1%)		
Missing	19	-		
Smoking status in the first trimester of pregnancy				
Smoker or quit	48	(21.3%)		
Never	177	(78.7%)		
Missing	17	-		
Smoking status in the third trimester of pregnancy				
Smoker or quit	52	(22.7%)		
Never	177	(77.3%)		
Missing	13	-		
*FADS1*; rs174547 genotype				
TT	97	(40.1%)		
TC	108	(44.6%)		
CC	37	(15.3%)		
Infants				
Gestational period, day	226		278	(271–284)
Sex				
Male	117	(52.2%)		
Female	107	(47.8%)		
Missing	18			
*FADS1*; rs174547 genotype				
TT	80	(35.2%)		
TC	113	(49.8%)		
CC	34	(15.0%)		
Missing	15	-		

-, Percent is not calculated for missing.

**Table 2:** Fatty acid compositions of maternal plasma glycerophospholipids according to gestational age ^1^.

	The 1st Trimester		The 3rd Trimester		At Delivery		*p* ^2^
	n = 242		n = 237		n = 213		
SFA (%)	40.14	(39.01–41.20) ^a^	41.78	(40.28–42.76) ^b^	41.89	(40.88–42.82) ^b^	<0.001
MUFA (%)	18.18	(16.55–19.50) ^a^	18.48	(17.17–19.82) ^b^	18.60	(17.51–19.96) ^b^	0.001
PUFA (%)	41.90	(40.6–43.32) ^a^	40.02	(38.75–41.06) ^b^	39.46	(38.33–40.80) ^b^	<0.001
n−6 (%)							
18:2n−6 (LA)	22.91	(21.01–24.74)	22.99	(21.16–24.60)	22.93	(21.26–24.85)	0.943
18:3n−6 (GLA) ^3^	0.07	(0.05–0.11) ^a^	0.05	(0.03–0.07) ^b^	0.05	(0.04–0.08) ^c^	<0.001
20:3n−6 (DGLA)	2.13	(1.74–2.55) ^a^	2.40	(2.12–2.75) ^b^	2.44	(2.08–2.74) ^b^	<0.001
20:4n−6 (ARA)	8.76	(7.73–9.72) ^a^	7.07	(6.32–7.85) ^b^	6.92	(6.14–7.84) ^b^	<0.001
n−3 (%)							
18:3n−3 (ALA) ^3^	0.51	(0.40–0.64) ^a^	0.58	(0.47–0.70) ^b^	0.55	(0.47–0.64) ^b^	<0.001
20:5n−3 (EPA) ^3^	0.72	(0.52–1.08) ^a^	0.58	(0.38–0.95) ^b^	0.65	(0.39–1.00) ^b^	<0.001
22:5n−3 (DPA)	0.63	(0.54–0.76) ^a^	0.52	(0.42–0.62) ^b^	0.47	(0.37–0.56) ^c^	<0.001
22:6n−3 (DHA)	4.84	(4.16–5.30) ^a^	4.47	(3.75–5.23) ^b^	4.28	(3.50–5.01) ^c^	<0.001
Ratios							
ARA/LA ratio	0.37	(0.32–0.45) ^a^	0.31	(0.27–0.37) ^b^	0.30	(0.26–0.36) ^b^	<0.001
ARA/DGLA ratio ^3^	4.10	(3.31–4.93) ^a^	2.91	(2.56–3.38) ^b^	2.91	(2.57–3.30) ^b^	<0.001

SFA, saturated fatty acids; MUFA, monounsaturated fatty acids; PUFA, polyunsaturated fatty acids. ^1^ Values are presented as median (25th%–75th%). ^2^ Comparisons of fatty acid composition in serum glycerophospholipids by stages of pregnancy were performed using repeated one-way analysis of variance, followed by the Tukey–Kramer HSD test for multiple comparisons between groups. Values within a row with unlike superscript letters were significantly different (*p* < 0.05). ^3^ Data were converted to natural logarithms and then used for statistical analysis.

**Table 3:** Associations of maternal FADS1 gene polymorphisms with fatty acid compositions of maternal serum glycerophospholipids ^1^.

Objective Variable ^2^	The 1st Trimester			The 3rd Trimester			At Delivery		
	n = 185			n = 180			n = 165		
	B	Std β	*p*	B	Std β	*p*	B	Std β	*p*
SFA	−0.216	−0.090	0.233	−0.050	−0.019	0.801	−0.342	−0.165	0.036
MUFA	0.369	0.122	0.091	0.333	0.132	0.079	0.566	0.215	0.005
PUFA	−0.159	−0.052	0.469	−0.249	−0.094	0.207	−0.186	−0.064	0.410
n−6									
18:2n−6 (LA)	1.316	0.351	<0.001	1.179	0.348	<0.001	1.289	0.395	<0.001
18:3n−6 (GLA)	−0.449	−0.466	<0.001	−0.232	−0.271	<0.001	−0.326	−0.360	<0.001
20:3n−6 (DGLA)	−0.176	−0.216	0.003	−0.144	−0.212	0.004	−0.098	−0.154	0.051
20:4n−6 (ARA)	−1.089	−0.491	<0.001	−0.953	−0.636	<0.001	−0.926	−0.562	<0.001
n−3									
18:3n−3 (ALA)	0.116	0.223	0.003	0.098	0.231	0.002	0.036	0.157	0.047
20:5n−3 (EPA)	−0.159	−0.222	0.002	−0.127	−0.142	0.052	−0.133	−0.141	0.063
22:5n−3 (DPA)	−0.015	−0.061	0.414	−0.001	−0.003	0.970	0.005	0.025	0.745
22:6n−3 (DHA)	−0.108	−0.074	0.321	−0.278	−0.193	0.008	−0.307	−0.208	0.006
Ratios									
ARA/LA ratio	−0.069	−0.498	<0.001	−0.059	−0.626	<0.001	−0.057	−0.581	<0.001
ARA/DGLA ratio	−0.116	−0.063	0.374	−0.204	−0.219	0.003	−0.228	−0.254	0.001

^1^ Statistical analysis was performed with multiple regression analysis using fatty acid composition as the objective variable and rs174547 polymorphism as the explanatory variable. The covariates were corresponding fatty acid intake, mother’s age, mother’s non-pregnant BMI, and smoking. ^2^ The following variables were used in the analysis after natural log transformation: ALA, EPA, and GLA at the 1st trimester; ALA, EPA, and GLA at the 3rd trimester; and EPA and GLA at delivery.

## Data Availability

The data sets used and analyzed in this study are available from the C-MACH research committee upon reasonable request.
